# Association of a novel index, the total cholesterol–high-density lipoprotein–glucose index, with diabetes mellitus in Chinese adults: a retrospective cohort study

**DOI:** 10.3389/fendo.2026.1774965

**Published:** 2026-04-01

**Authors:** Yajing Gao, Chuang Gao, Jiaqian Zhu, Yong Han

**Affiliations:** 1 Department of Anesthesiology, Shenzhen Maternity and Child Healthcare Hospital, Women and Children's Medical Center, Southern Medical University, Shenzhen, Guangdong, China; 2 Department of Emergency, Shenzhen Dapeng New District Kuichong People’s Hospital, Shenzhen, Guangdong, China; 3 Department of Neurology, Yiwu Central Hospital, Yiwu, Zhejiang, China; 4 School of Medicine, Shenzhen University, The First Affiliated Hospital of Shenzhen University, Shenzhen Second People’s Hospital, Shenzhen, Guangdong, China; 5 Department of Emergency, Shenzhen Second People’s Hospital, The First Affiliated Hospital of Shenzhen University, Shenzhen, Guangdong, China

**Keywords:** CHG index, diabetes mellitus, dyslipidemia, glucose metabolism abnormalities, predictive value

## Abstract

**Objective:**

Research on the relationship between Total Cholesterol, High-Density Lipoprotein, and Glucose (CHG) index and diabetes mellitus (DM) risk remains limited. This study aims to investigate the association between CHG and DM incidence.

**Methods:**

This retrospective cohort study included 8,844 participants who underwent comprehensive health examinations at Shenzhen Kuichong People’s Hospital between 2018 and 2023. The maximum individual follow-up duration reached 5 years, with a median (interquartile range) follow-up period of 2.72 (2.58–4.78) years. Cox proportional hazards regression models were used to evaluate the association between CHG and DM risk. ROC analysis was conducted to evaluate the predictive capability of CHG for DM. Finally, subgroup analyses and sensitivity analyses were performed to further verify the stability of these findings.

**Results:**

After multivariate adjustment, the CHG index was independently associated with increased DM risk, with an HR of 1.150 (95% CI: 1.098–1.204) per 0.1 unit increase in CHG. Furthermore, ROC curve analysis demonstrated that CHG had the highest AUC (0.7377) for predicting DM risk, compared to TC (0.5375), HDL-c (0.6105), and FPG (0.6761). The stability of these results was further validated through sensitivity and subgroup analyses.

**Conclusion:**

This study found an independent positive relationship between CHG and DM risk. Additionally, CHG demonstrates a certain predictive value for DM risk. This helps clinicians identify high-risk individuals for DM early and provides a new perspective for optimizing clinical prevention and management of DM.

## Introduction

Diabetes mellitus (DM) is a group of metabolic disorders characterized by chronic hyperglycemia caused by various etiologies, resulting from defects in insulin secretion and/or action (1). According to the International Diabetes Federation Diabetes Atlas, approximately 425 million people worldwide had DM in 2017, and this number is projected to rise to 629 million by 2045 (2). Furthermore, DM can lead to multiple complications affecting the kidneys, nervous system, eyes, and cardiovascular system (3–6), and has become one of the leading causes of disability and death (7). With the continuously rising prevalence of DM and its increasing mortality burden, this disease has emerged as a major challenge facing global health systems (3, 8, 9). Therefore, identifying DM risk factors and implementing targeted preventive interventions is of great clinical significance for reducing the incidence of DM and its complications.

Dyslipidemia is characterized by decreased high-density lipoprotein cholesterol (HDL-c) levels with concurrent elevations in total cholesterol (TC), triglycerides (TG), and low-density lipoprotein cholesterol (LDL-c) concentrations, which significantly increases the risk of DM and cardiovascular and cerebrovascular events (10–12). Multiple studies have demonstrated that elevated TC or reduced HDL-c levels are significantly positively correlated with DM incidence (12–14). At the same time, research confirms that the ratio of TC to HDL-c is closely associated with insulin resistance (IR), DM, and cardiovascular mortality (15–18). Additionally, research indicates that even when fasting plasma glucose (FPG) values fall within the normal range, individuals with levels approaching the upper normal limit (e.g., 4.50-5.50 mmol/L) still exhibit a higher risk of DM compared to those with lower values (19). In addition, elevated TC promotes pathological intracellular cholesterol accumulation in hepatocytes and skeletal myocytes, which impairs insulin signaling and exacerbates IR (20, 21). Concurrently, reduced HDL-c diminishes Apolipoprotein A-I(ApoA-I) availability and impairs ATP-Binding Cassette Transporter A1(ABCA1)-mediated reverse cholesterol transport, reinforcing intracellular cholesterol retention and further aggravating IR (22, 23). In this lipotoxic environment, even modest elevations in FPG may reflect early pancreatic β-cell compensatory failure (24). Therefore, TC, HDL-c, and FPG do not act as independent parallel risk factors for DM, but rather converge upon a synergistic pathological state characterized by cholesterol-driven IR, impaired reverse cholesterol transport, and lipotoxic β-cell decompensation (25). We therefore hypothesize that a composite index integrating TC, HDL-c, and FPG may more comprehensively capture this complex pathophysiological process, thereby providing superior information for DM risk assessment and early prediction.

In recent years, Amin Mansoori and colleagues have proposed a novel index called Total Cholesterol, High-Density Lipoprotein, and Glucose (CHG) index, which comprises TC, HDL-c, and FPG (26). Studies have demonstrated that the CHG index shows significant clinical value in predicting microvascular complications of DM(such as retinopathy and nephropathy) and assessing cardiovascular event risk (27, 28). However, research on the association between the CHG index and DM remains relatively limited, with only two cross-sectional studies having addressed this topic. Cross-sectional evidence from a Chinese population (using China Health and Retirement Longitudinal Study-2011 baseline data, among adults aged ≥45 years) and a U.S. population (the National Health and Nutrition Examination Survey, NHANES 2009–2018) has supported a positive association between CHG and prevalent DM, with AUC values of 0.825 and 0.721, respectively (29, 30). Nevertheless, cross-sectional designs cannot address the temporal relationship between CHG and future DM risk. Additionally, considering the differences in implementation time, CHG ranges, sex ratios, and adjustment factors across studies, the relationship between CHG and DM risk in the Chinese population remains unclear. Therefore, we conducted a retrospective cohort study aimed at exploring the association between the CHG index and DM risk, and further evaluating the predictive value of the CHG index for DM.

## Methods

### Study design and study population

This study employed a retrospective cohort design. The study subjects were individuals who underwent routine physical examinations at Kuichong People’s Hospital in Dapeng New District, Shenzhen, from January 2018 to December 2023. CHG was used as the independent variable, while DM served as the dependent variable (using binary coding: 0 representing non-DM, 1 representing DM).

**Figure 1:**
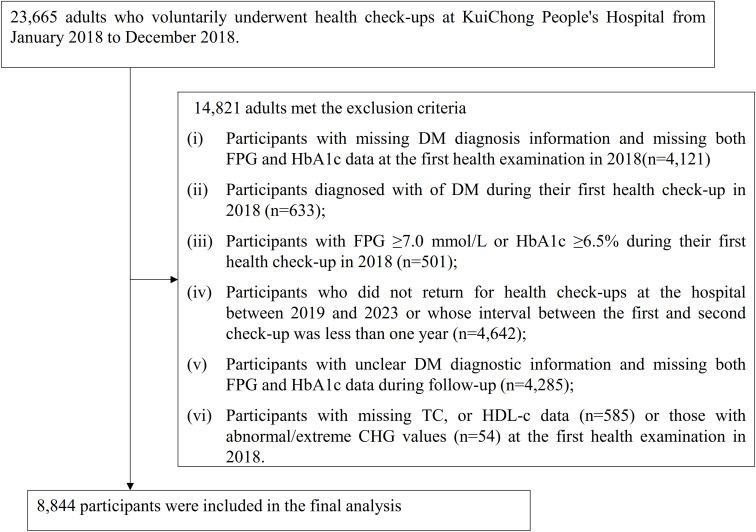
Flowchart illustrating the study participants.

The initial study population included 23,665 individuals aged 18 years and above who completed routine physical examinations between January and December 2018. Participants were excluded based on the following criteria: (i) those lacking DM diagnosis information and simultaneously missing FPG and HbA1c data at their first physical examination in 2018 (n=4,121); (ii) those diagnosed with DM at the initial health assessment in 2018 (n=633); (iii) those with FPG ≥7.0 mmol/L or hemoglobin A1c (HbA1c) ≥6.5% at the baseline assessment in 2018 (n=501); (iv) subjects who did not return for physical examination between 2019 and 2023 or whose interval between first and second physical examinations was less than one year (n=4,642); (v) those with unclear DM diagnosis results and missing FPG and HbA1c data during follow-up assessments (n=4,285); (vi) those missing TC, HDL-c data (n=585) or with abnormal/extreme CHG values (n=54). The final analysis cohort included 8,844 subjects. The participant enrollment process is detailed in **Figure 1**.

### Ethical approval and consent

This study obtained ethical approval from the Ethics Committee of Kuichong People’s Hospital in Dapeng New District, Shenzhen (approval number: 2024005). The requirement for informed consent was formally waived by the Ethics Committee due to the retrospective nature of the investigation and the use of completely de-identified subject information. The research was conducted in strict accordance with the principles outlined in the Declaration of Helsinki and complied with all applicable ethical guidelines and requirements.

### Variables

#### The total cholesterol, high-density lipoprotein, and glucose index

CHG index was evaluated as a continuous measure. The index was computed according to the formula: CHG= Ln[(TC × FPG)/(2 × HDL-c)], where TC, FPG, and HDL-c were measured in mg/dl (26).

#### Diagnosis of DM and Follow-up

Incident DM was defined as: participants who were free of DM at baseline in 2018 and subsequently reported having DM during the follow-up period. The diagnostic criteria were met if any of the following conditions were present: self-reported physician-diagnosed DM during follow-up, FPG ≥7.0 mmol/L, or HbA1c ≥6.5% (31).

Information regarding DM history was collected through standardized questionnaires administered at each follow-up visit, which inquired whether subjects had been diagnosed with DM by a physician, the date of diagnosis, and if they had received glucose-lowering treatment. The cohort was established based on baseline examinations conducted between January and December 2018, with follow-up information obtained from subsequent routine health examinations and questionnaire records during 2019–2023; the administrative end of follow-up was defined as the date of the last available follow-up record in the database in 2023. The time to incident DM was defined as the interval from baseline to the date on which a participant first met the diagnostic criteria for DM. For participants who remained DM-free, follow-up time was calculated from baseline to the date of their last available follow-up examination, at which point they were censored.

#### Covariates

The selection of covariates was based on our clinical experience and findings from previous studies (12, 15, 26). Covariates included in this study were: (i) Continuous variables: age, Systolic Blood Pressure (SBP), Diastolic Blood Pressure (DBP), Body Mass Index (BMI), Waist Circumference (WC), TC, LDL-c, HDL-c, TG, FPG, Aspartate Aminotransferase (AST), Alanine Aminotransferase (ALT), C-Reactive Protein (CRP), Gamma-Glutamyl Transferase (GGT), Hemoglobin A1c (HBA1C), Serum creatinine (Scr). (ii) Categorical variables: sex, smoking status, Dyslipidemia (DLP), Hypertension (HTN), antihypertensive medication (HTN-MED), antihyperlipidemic medication (DLP-MED), drinking status, and physical activity.

### Data collection

Professionally trained clinical staff conducted systematic measurement assessments of all participants. The research team used standardized questionnaires to comprehensively collect baseline characteristic data, including sociodemographic characteristics (such as sex, age), behavioral factors (such as smoking and physical activity), and medical history (such as HTN, DLP, and medication status) and other important information. Blood pressure measurements were performed using standard mercury sphygmomanometers, strictly following clinical protocols. Before all biochemical indicator testing, participants were required to fast for at least 10 hours, after which venous blood samples were collected by professional medical staff. A Beckman 5800 fully automated analyzer was used to test blood samples for TC, TG, HDL-c, LDL-c, and FPG.

### Handling of missing data

Missing data is a common and unavoidable situation in observational studies. In this analysis, multiple variables had missing values, including drinking status (576, 6.51%), smoking status (302, 3.41%), WC (9, 0.10%), DBP (25, 0.28%), SBP (25, 0.28%), DLP (131, 1.48%), Scr (119, 1.35%), AST (143, 1.62%), ALT (143, 1.62%), and GGT (75, 0.85%). To minimize potential bias caused by missing data, we used multiple imputation to fill in missing values in the dataset (32, 33). This method was based on linear regression, completed after 10 iterations, incorporating variables including age, SBP, DBP, BMI, WC, LDL-c, TG, AST, ALT, CRP, GGT, HBA1c, Scr, sex, smoking status, DLP, HTN, HTN-MED, DLP-MED, drinking status, and physical activity. In accordance with established analytical standards, missing values were assumed to be Missing at Random (MAR) (33).

### Statistical analysis

Participants were stratified and compared according to CHG quartiles. Normally distributed continuous variables were presented as mean ± standard deviation (SD), while non-normally distributed data were described using median and interquartile range (IQR). Categorical variables were expressed as frequencies and their corresponding percentages. Comparisons of continuous variables between groups were performed using analysis of variance (ANOVA) or Kruskal-Wallis test, while categorical variables were compared using chi-square test (χ²).

This study employed univariate and multivariate Cox proportional hazards regression models to explore the association between CHG and DM risk. Importantly, prior to the analysis, the proportional hazards assumption (PH) was evaluated using Schoenfeld residuals. The global test and CHG-specific test both yielded P-values > 0.05, confirming the validity of Cox proportional hazards regression. In addition, prior to model construction, variance inflation factors (VIFs) were calculated for all candidate covariates to assess multicollinearity, with VIF > 5 being used as the threshold to indicate significant collinearity. Three models were constructed: (i) Model I: unadjusted for any covariates; (ii) Model II: adjusted for sex, age, BMI, smoking and drinking status; (iii) Model III: adjusted for age, BMI, Scr, drinking status, ALT, TG, HbA1C, physical activity, DBP, smoking status, AST, HTN, DLP-MED, and SBP. Adjustment variables were selected based on previous studies and clinical experience. Due to multicollinearity with other predictors, WC was excluded from the multivariate models (**Supplementary Table 1**).

Previous studies have confirmed significant associations between HTN, obesity, smoking, and glucose metabolism (34–36). To verify the robustness of our findings, we conducted sensitivity analyses by separately **e**xcluding subjects with BMI≥28 kg/m² (37), smokers, and patients diagnosed with HTN. Additionally, in the multivariate Cox regression models, we introduced Generalized Additive Models (GAM) to incorporate continuous covariates as smooth curves in the analysis. Finally, we calculated E-values to assess the potential influence of unmeasured and unknown confounding variables on the association between CHG and DM risk (38).

Subgroup analyses were conducted using stratified Cox regression models, with stratification based on age, sex, SBP, DBP, physical activity, smoking, and drinking status. According to clinical thresholds, the following variables were categorized: age was divided into four groups: <40 years, 40–50 years, and ≥50 years; SBP was categorized into two groups: <140 mmHg or ≥140 mmHg; DBP was divided into two groups: <90 mmHg or ≥90 mmHg (39). The covariate adjustment models included variables such as age, BMI, drinking status, ALT, TG, HbA1c, physical activity, DBP, smoking status, AST, HTN, DLP-MED, and SBP, while excluding the stratification variables. Log-likelihood ratio tests were used to compare models with and without interaction terms to evaluate potential interactive effects.

Finally, ROC curves were constructed to evaluate the predictive ability of TC, FPG, HDL-c, and CHG for DM risk. The corresponding area under the curve (AUC), best thresholds, sensitivity, and specificity were also calculated. Pairwise comparisons of AUCs were performed using DeLong’s test for correlated ROC curves, with P < 0.05 indicating statistical significance.

Results were reported in accordance with the STROBE statement guidelines (40). Statistical analyses were conducted using R software (v3.4.3) and Empower (v4.2). A two-sided P value <0.05 was considered statistically significant.

## Results

### Participant characteristics

**Figure 2:**
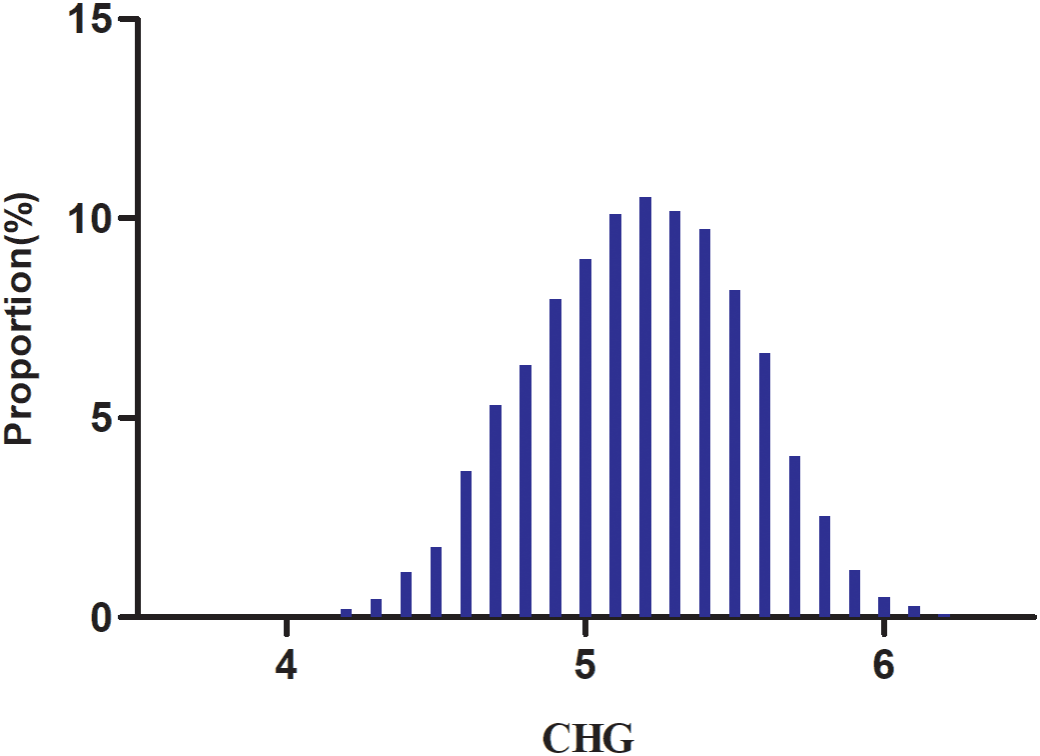
Distribution of CHG. The distribution approximated a normal distribution, spanning from 4.16 to 6.23, with a mean ± SD of 5.18 ± 0.35.

**Table 1** presents the demographic and clinical characteristics of 8,844 study participants, with male subjects accounting for 74.92% of the total population. As shown in **Figure 2**, CHG distribution exhibited a normal distribution, ranging from 4.16 to 6.23, with a mean ± standard deviation (SD)value of 5.18 ± 0.35. Participants were divided into four groups according to CHG quartiles: Q1 group (≤4.93), Q2 group (4.93-5.19), Q3 group (5.19-5.44), and Q4 group (≥5.44). Compared to the Q1 group, participants in higher quartiles had elevated levels of age, SBP, DBP, BMI, WC, TC, LDL-c, TG, FPG, AST, ALT, CRP, GGT, and HBA1c, while HDL-c levels were relatively lower. Additionally, higher quartile groups had greater proportions of males and individuals with DLP, HTN, HTN-MED, DLP-MED, current drinking and sedentary compared to the Q1 group.

**Table 1:** Baseline characteristics of participants.

CHG quartiles	Q1(≤4.93)	Q2(4.93-5.19)	Q3(5.19-5.44)	Q4(≥5.44)	P-value
N	2210	2212	2211	2211	
Age(years)	39.68 ± 8.44	41.96 ± 8.85	42.72 ± 8.50	42.91 ± 8.17	<0.001
SBP (mmHg)	110.69 ± 11.30	116.36 ± 11.65	119.04 ± 11.78	122.01 ± 12.69	<0.001
DBP (mmHg)	72.03 ± 7.61	75.67 ± 7.59	77.43 ± 7.57	79.34 ± 7.88	<0.001
BMI (kg/m^2^)	23.86 ± 3.30	25.72 ± 3.50	26.73 ± 3.58	28.04 ± 3.85	<0.001
WC (cm)	82.43 ± 10.93	90.12 ± 11.09	94.28 ± 10.27	98.13 ± 10.45	<0.001
TC (mg/dL)	173.90 ± 29.61	188.25 ± 29.78	202.86 ± 31.02	224.70 ± 35.51	<0.001
LDL-c(mg/dL)	94.23 ± 22.51	116.98 ± 23.78	132.61 ± 26.62	149.39 ± 33.21	<0.001
HDL-c(mg/dL)	63.12 ± 13.01	50.99 ± 8.69	44.26 ± 7.07	37.47 ± 6.49	<0.001
TG (mg/dL)	74.00 (59.00-97.00)	92.00 (73.00-120.00)	122.00 (94.00-156.00)	172.00 (131.00-226.50)	<0.001
FPG (mg/dL)	81.11 ± 6.90	85.79 ± 7.28	88.35 ± 7.47	92.29 ± 8.82	<0.001
AST(u/L)	26.28 ± 11.64	28.78 ± 9.27	30.19 ± 12.59	32.00 ± 11.95	<0.001
ALT(u/L)	28.00 (22.00-36.00)	34.00 (27.00-44.00)	38.00 (30.00-50.00)	43.00 (33.00-57.00)	<0.001
CRP (mg/dL)	0.90 (0.40-2.20)	1.00 (0.50-2.10)	1.20 (0.60-2.40)	1.60 (0.80-2.90)	<0.001
GGT(u/L)	20.00 (15.00-27.00)	24.00 (18.00-35.00)	28.00 (22.00-41.00)	35.00 (27.00-49.00)	<0.001
HBA1C (%)	5.12 ± 0.21	5.26 ± 0.22	5.34 ± 0.23	5.46 ± 0.27	<0.001
Scr (umol/L)	69.07 ± 15.39	70.04 ± 15.24	69.92 ± 15.42	70.58 ± 15.82	0.129
Sex (n, %)					<0.001
Female	1344 (60.81%)	542 (24.50%)	221 (10.00%)	111 (5.02%)	
Male	866 (39.19%)	1670 (75.50%)	1990 (90.00%)	2100 (94.98%)	
Smoking (n, %)	140 (6.33%)	140 (6.33%)	178 (8.05%)	228 (10.31%)	<0.001
DLP (n, %)	276 (12.49%)	537 (24.28%)	708 (32.02%)	910 (41.16%)	<0.001
HTN (n, %)	118 (5.34%)	225 (10.17%)	247 (11.17%)	303 (13.70%)	<0.001
HTN-MED (n, %)	120 (5.43%)	228 (10.31%)	246 (11.13%)	291 (13.16%)	<0.001
DLP-MED (n, %)	156 (7.06%)	259 (11.71%)	204 (9.23%)	151 (6.83%)	<0.001
Physical Activity (n, %)					<0.001
Sedentary	410 (18.55%)	403 (18.22%)	486 (21.98%)	616 (27.86%)	
Light activity	791 (35.79%)	834 (37.70%)	865 (39.12%)	867 (39.21%)	
Moderate activity	763 (34.52%)	732 (33.09%)	696 (31.48%)	613 (27.73%)	
Vigorous activity	246 (11.13%)	243 (10.99%)	164 (7.42%)	115 (5.20%)	
Drinking status					0.227
Ever	1845 (83.48)	1796 (81.19)	1779 (80.46)	1767 (79.92)	
Current	277 (12.54)	317 (14.33)	330 (14.93)	335 (15.15)	
Never	88 (3.98)	99 (4.48)	102(4.61)	109(8.59)	

Continuous variables were presented either as mean ± standard deviation or as median with interquartile range, depending on data distribution. Categorical data were reported as counts and percentages. BMI, Body Mass Index; WC, Waist Circumference; TC, Total Cholesterol; LDL-c, Low-Density Lipoprotein Cholesterol; HDL-c, High-Density Lipoprotein Cholesterol; TG, Triglycerides; FPG, Fasting Plasma Glucose; AST, Aspartate Aminotransferase; ALT, Alanine Aminotransferase; CRP, C-Reactive Protein; GGT, Gamma-Glutamyl Transferase; HbA1c, Hemoglobin A1c; Scr, Serum Creatinine; DLP, Dyslipidemia; HTN, Hypertension; HTN-MED, Hypertension Medication; N, Number; DLP-MED, Dyslipidemia Medication.

### The incidence of DM

During a median follow-up of 2.72 (IQR: 2.58–4.78) years, 324 incident cases of DM (3.66%) were identified among the 8,844 participants. The cohort was established based on baseline examinations conducted in 2018, with subsequent follow-up examinations performed between 2019 and 2023; thus, the observation window spanned up to 5 years, while individual follow-up durations varied due to differences in return visit timing and censoring. After stratification by CHG quartiles, the incidence rates of DM (per 10,000 person-years) were: 104.11 for Q1, 179.34 for Q2, 308.81 for Q3, and 485.62 for Q4. The overall cumulative DM incidence was 3.66%, with specific incidence rates by quartile as follows: 1.40% in Q1, 2.40% in Q2, 4.21% in Q3, and 6.65% in Q4. Notably, compared to the Q1 group (lowest CHG level), the Q4 group (highest CHG level) demonstrated significantly higher DM incidence (P for trend <0.001) (**Table 2**).

**Table 2:** Incidence rate of DM (% or Per 10000 person-year).

CHG quartiles	Participants(n)	DM events(n)	Incidence rate (95% CI) (%)	Per 10,000 person-year
Total	8844	324	3.66(3.27 -4.06)	269.63
Q1	2210	31	1.40 (0.91-1.89)	104.11
Q2	2212	53	2.40 (1.76-3.03)	179.34
Q3	2211	93	4.21 (3.37-5.04)	308.81
Q4	2211	147	6.65 (5.61-7.69)	485.62
P for trend			<0.001	

CI, confidence; n, number.

**Figure 3:**
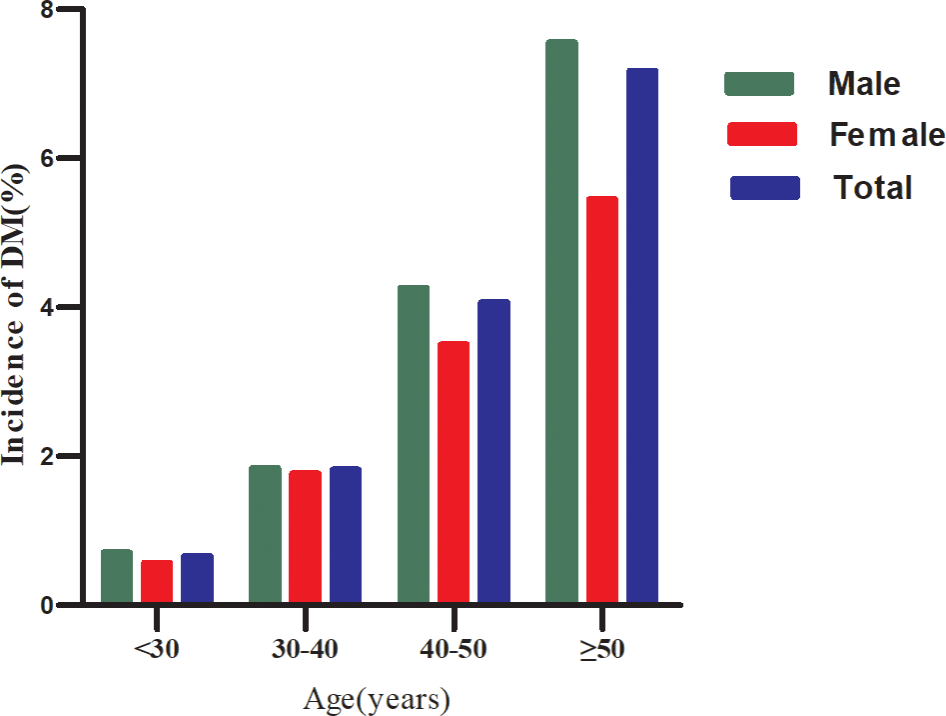
Diabetes incidence (%) Stratified by age and sex.

Age was divided into four groups: <30 years, 30–40 years, 40–50 years, and ≥50 years. Males had a higher risk of DM than females in all age groups (**Figure 3**). Furthermore, regardless of sex, the incidence of DM increased with age.

**Figure 4:**
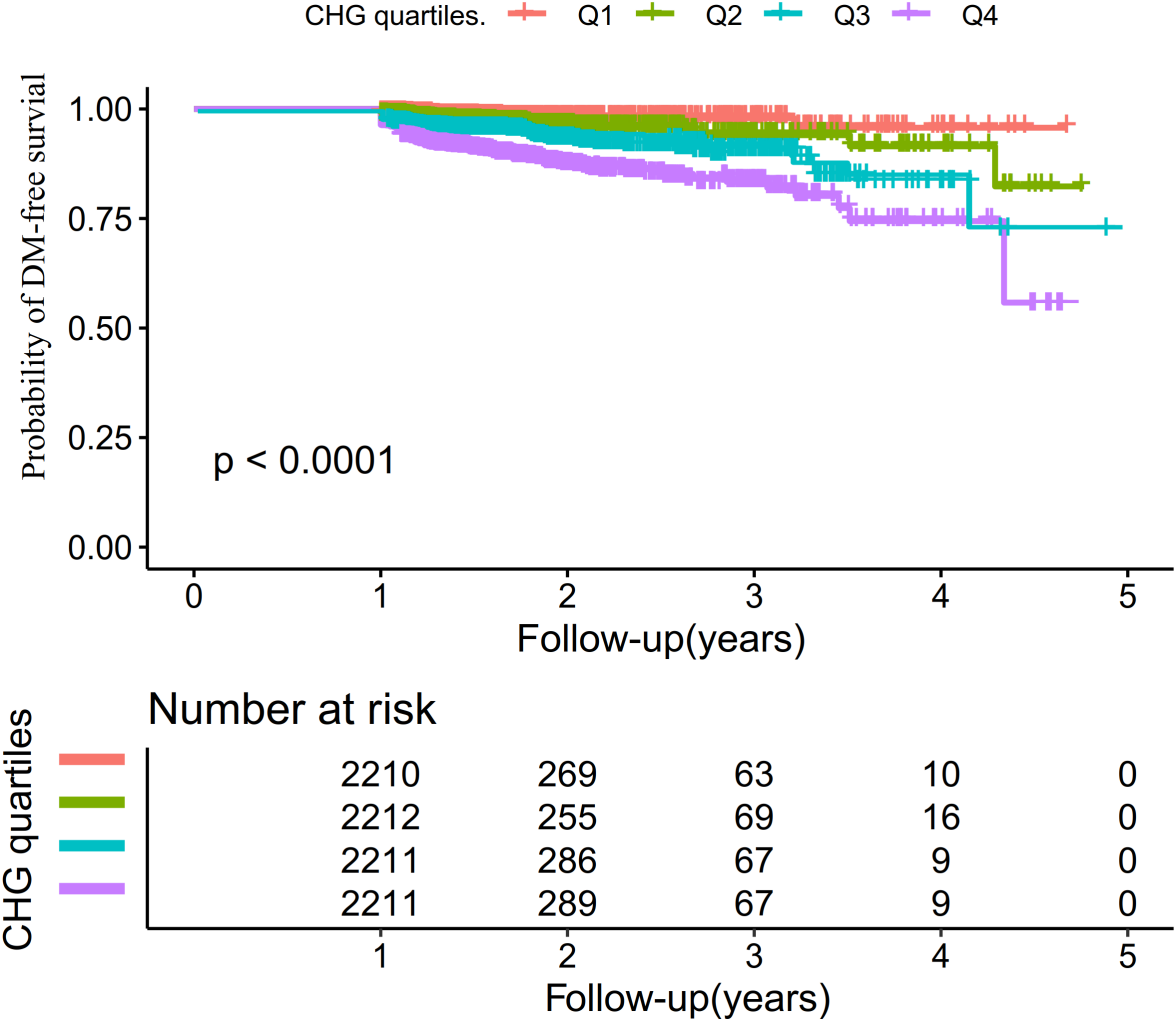
Kaplan-Meier curves illustrating DM–free survival probabilities across CHG quartiles.

The Kaplan-Meier survival curves stratified by CHG quartiles (**Figure 4**) showed the probability of DM-free survival. There were significant differences in DM-free survival rates among different CHG quartile groups (log-rank test, P < 0.001). Analysis indicated that participants in the highest CHG quartile group had the highest risk of DM.

### Factors influencing the risk of DM analyzed by univariate Cox proportional hazards regression

Univariate analysis results showed that DM risk was significantly positively associated with age, DBP, BMI, SBP, WC, TC, TG, FPG, ALT, AST, HBA1c, Scr, and CHG levels, while HDL-c levels were significantly negatively associated with DM risk. Additionally, males and individuals with DLP, HTN, HTN-MED, DLP-MED, and Sedentary lifestyle were more likely to develop DM. However, LDL-c, CRP levels, and smoking status showed no significant association with DM risk (**Supplementary Table 2**).

### The relationship between CHG and the risk of DM

Three Cox proportional hazards regression models were constructed to explore the relationship between CHG and DM risk. The unadjusted Model I showed that for each 0.1 unit increase in CHG, DM risk increased by 19.6% (HR = 1.196, 95% CI: 1.157-1.236). In Model II, after adjusting for sex, age, BMI, smoking, and drinking status, each 0.1 unit increase in CHG was associated with a 17.1% higher risk of DM (HR = 1.171, 95% CI: 1.121-1.224). The fully adjusted Model III demonstrated that this association remained statistically significant: each 0.1 unit increase in CHG elevated DM risk by 16% (HR = 1.150, 95% CI: 1.098-1.204) (**Table 3**).

**Table 3:** Association of CHG with DM risk using various models.

Exposure	Model I(HR,95%CI) P	Model II(HR,95%CI) P	Model III(HR,95%CI) P	Model IV(HR,95%CI) P
CHG (per 0.1-unit)	1.196 (1.157, 1.236) <0.001	1.171 (1.121, 1.224) <0.001	1.150 (1.098, 1.204) <0.001	1.154 (1.099, 1.213) <0.00001
CHG quartiles				
Q1	Ref	Ref	Ref	Ref
Q2	1.726 (1.108, 2.688) 0.0158	1.639 (1.035, 2.596) 0.035	1.325 (0.838, 2.098) 0.229	1.240 (0.770, 1.996) 0.376
Q3	2.948 (1.964, 4.427) <0.001	2.428 (1.546, 3.813) <0.001	1.966 (1.257, 3.075) 0.003	1.918 (1.182, 3.111) 0.008
Q4	4.578 (3.108, 6.745) <0.001	3.274 (2.072, 5.172) <0.001	2.420 (1.512, 3.876) <0.001	2.366 (1.418, 3.946) <0.001
P for trend	<0.001	<0.001	<0.001	<0.001

CI, confidence, Re, reference; HR, Hazard ratios. Model I: unadjusted for any covariates; Model II: adjusted for sex, age, BMI, smoking and drinking status; Model III: adjusted for age, BMI, Scr, drinking status, ALT, TG, HbA1C, physical activity, DBP, smoking, AST, HTN, DLP-MED, and SBP. Model IV: adjusted for age(smooth), BMI (smooth), drinking status, ALT (smooth), Scr(smooth), TG(smooth), HbA1c (smooth), physical activity, DBP (smooth), smoking, AST (smooth), HTN, DLP-MED, and SBP(smooth). HR, hazard ratio; Ref, reference; CI, confidence.

Additionally, after stratification by CHG quartiles, the data were re-entered into the Cox regression model. With Q1 as the reference group, multivariate adjusted analysis revealed: HR for Q2 was 1.325 (95% CI: 0.838, 2.098), for Q3 was 1.966 (95% CI:1.257, 3.075), and for Q4 was 2.420 (95% CI: 1.512, 3.876). These results indicated that compared to Q1, the risk of DM increased by 38% in Q2 without statistical significance, while the risk increased by 116.2% and 171.3% in Q3 and Q4, respectively (**Table 3**, Model III).

### Sensitivity analysis

To assess the stability of the research findings, various sensitivity analyses were conducted. Initially, a GAM approach was employed to incorporate continuous covariates as smooth curves into the model. The results obtained from this method were largely consistent with the fully adjusted **Table 3** Model III. Specifically, for each 0.1 unit increase in CHG, DM incidence increased by 15.4% (HR = 1.154, 95% CI: 1.099-1.213) (**Table 3**, Model IV). Furthermore, in a sensitivity analysis restricted to participants with BMI lower than 28 kg/m², after adjusting for confounding factors, the association between each 0.1 unit increase in CHG and DM risk remained significant (HR = 1.159, 95% CI: 1.087-1.235). After excluding HTN participants, the results remained consistent: for each 0.1 unit increase in CHG, the HR for DM was 1.134 (95% CI: 1.074-1.199). Finally, among non-smokers, this association maintained statistical significance, with an HR of 1.144(95% CI: 1.091-1.200) (**Table 4**).

**Table 4:** Association of CHG with DM risk across various sensitivity analyses.

Exposure	Model I(HR,95%CI) P	Model II(HR,95%CI) P	Model III(HR,95%CI) P
CHG (per 0.1-unit)	1.159 (1.087, 1.235) <0.001	1.134 (1.074, 1.199) <0.001	1.144 (1.091, 1.200) <0.001
CHG quartiles			
Q1	Ref	Ref	Ref
Q2	1.465 (0.815, 2.631) 0.202	1.171 (0.707, 1.939) 0.541	1.320 (0.827, 2.108) 0.245
Q3	2.713 (1.531, 4.807) <0.001	1.676 (1.023, 2.748) 0.040	1.974 (1.252, 3.112) 0.003
Q4	3.119 (1.674, 5.813) <0.001	2.036 (1.202, 3.446) 0.008	2.425 (1.498, 3.924) <0.001
P for trend	<0.001	0.002	<0.001

Model I was a sensitivity analysis among individuals with BMI values under 28 kg/m² (n=7,069). Age, drinking status, ALT, Scr, TG, HbA1C, physical activity, DBP, smoking status, AST, HTN, DLP-MED, and SBP were adjusted.

Model II conducted sensitivity analysis following the exclusion of HTN individuals (N = 7,842). Age, BMI, drinking status, ALT, TG, HbA1C, Scr, physical activity, DBP, smoking status, AST, DLP-MED, and SBP were adjusted.

Model III conducted sensitivity analysis following the exclusion of smokers (N = 8,043). Age, BMI, drinking status, ALT, Scr, TG, HbA1C, physical activity, DBP, AST, HTN, DLP-MED, and SBP were adjusted.

Furthermore, the E-value was 1.59, which was higher than the relative risk of association between CHG and potential unmeasured confounding factors (1.39), but lower than the relative risk of association between unmeasured confounding factors and DM (1.97). This suggests that unknown or unmeasured confounding factors were unlikely to significantly impact the association between CHG and DM risk. These sensitivity analyses enhanced the credibility and robustness of our research findings.

### Subgroup analysis

In both prespecified and exploratory subgroup analyses, we did not observe significant interaction effects between CHG and multiple covariates including age, sex, SBP, DBP, physical activity, DLP, and drinking status (all P for interaction >0.05). These findings suggest that the aforementioned factors did not significantly influence or modify the association between CHG and DM risk (**Supplementary Table 3**). The sample size and number of incident DM events per subgroup are presented in **Supplementary Table 3**; the number of DM events exceeded 20 in almost all subgroups, supporting adequate statistical power for stable estimates.

### ROC analysis of the predictive value of CHG, TC, FPG, and HDL-c for DM risk

**Figure 5:**
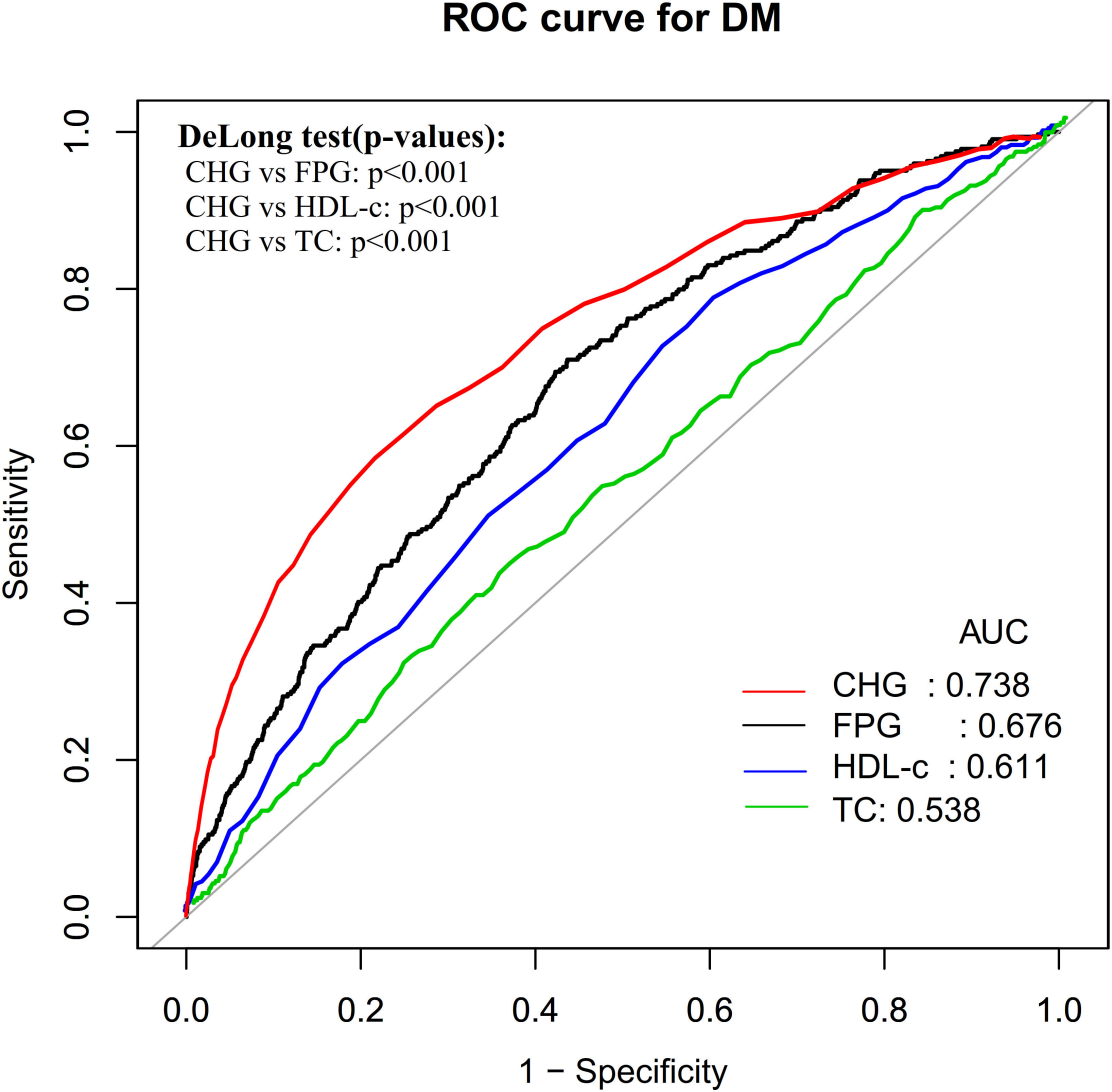
ROC curves for CHG, TC, FPG, and HDL-c prediction of DM.

ROC curves were plotted to evaluate the predictive ability of CHG, TC, FPG, and HDL-c for future DM risk (**Figure 5**). The AUC values for each variable were ranked as follows: TC: 0.5375 < HDL-c: 0.6105 < FPG: 0.6761 < CHG: 0.7377. The Youden index values for FPG, HDL-c, TC, and CHG were 0.2733, 0.1753, 0.0698, and 0.3624, respectively, corresponding to optimal cutoff values of 5.1944, 50.5000, 207.5000, and 5.2366 (**Table 5**). Pairwise DeLong’s tests demonstrated that the AUC of CHG was significantly higher than that of FPG, HDL-c, and TC (all P < 0.05), indicating that among the four indicators, CHG possesses stronger predictive capability for DM risk.

**Table 5:** The predictive value of CHG, TC, FPG, and HDL-c for DM risk.

Exposure	AUC (95%CI)	Best threshold	Specificity	Sensitivity	Youden index
FPG	0.6761(0.6473-0.7049)	5.1944	0.5634	0.7099	0.2733
HDL-c	0.6105(0.5798-0.6412)	50.5000	0.3944	0.7809	0.1753
TC	0.5375(0.5051-0.5700)	207.5000	0.6377	0.4321	0.0698
CHG	0.7377(0.7069-0.7685)	5.2366	0.8161	0.5463	0.3624

AUC, area under the curve; CHG, Total Cholesterol, High-Density Lipoprotein, and Glucose; TC, total cholesterol; FPG, fasting plasma glucose; HDL-c, high-density lipoprotein cholesterol.

## Discussion

This study demonstrates that CHG has an independent positive relationship with DM risk. Furthermore, ROC curve analysis indicates that CHG exhibits superior predictive performance for DM risk compared to other single indicators such as TC, FPG, and HDL-c.

Multiple studies have confirmed that lipid metabolism disorders are closely associated with significantly increased risks of DM (10, 12–14). In a prospective cohort study involving 117,268 participants, multivariate Cox regression analysis demonstrated that each standard deviation increase in the TC/HDL-c ratio was associated with a 27% increased risk of DM (HR 1.27, 95% CI: 1.09-1.48) (15). Another cross-sectional study based on 9,078 adults aged ≥18 years showed that TC/HDL-c was independently and positively associated with DM risk (HR: 1.42, 95% CI: 1.30–1.57) (41). Furthermore, abnormal glucose metabolism has been established as a key factor in the development of DM (42, 43). In a prospective cohort study including 3,981 participants without DM, the change in FPG (defined as follow-up FPG value minus baseline FPG value) was stratified by quartiles, and multivariate models showed that the highest quartile had a 65% increased risk of DM compared to the lowest quartile (HR: 1.65, 95% CI 1.2-2.27) (44). In another cohort study involving 13,163 men with normal baseline FPG levels (below 5.55 mmol/L), multivariate analysis revealed that individuals with FPG levels of 5.27-5.50 mmol/L had a HR of 2.84 (95% CI 1.67–4.87) for DM risk compared to those with levels of 2.78-4.50 mmol/L (19). Therefore, we hypothesized that compared with single indicators, the composite indicator CHG, which integrates TC, HDL-c, and FPG, might provide more comprehensive and precise information for DM risk assessment and early prediction. Previous studies have shown that the CHG index plays an important role in assessing the risk of cardiovascular events and in the diagnosis of diabetic retinopathy and nephropathy (27, 28). However, research on the association between the CHG index and DM risk is relatively scarce. A cross-sectional study using CHARLS 2011 baseline data (n=8,251, adults aged ≥45 years) found that for every one-unit increase in the CHG index, the prevalence of DM increased by 1.5 times (29). Another cross-sectional study based on NHANES data (2009–2018) also confirmed a significant association between elevated CHG levels and an increased prevalence of DM (OR: 4.30, 95% CI: 3.21-5.77) (30). The present study extends these cross-sectional findings by adopting a longitudinal cohort design with incident DM as the outcome. Importantly, our study cohort—comprising participants from a real-world health-examination setting with a broader age range—similarly supports an independent positive association between the CHG index and the risk of incident DM. Furthermore, we analyzed CHG as both a categorical variable and a continuous variable to minimize information loss and more accurately quantify its association with the outcome. We conducted sensitivity analyses separately on participants with BMI below 28 kg/m², without HTN, and those who had never smoked. The results confirmed the consistency of these findings across these specific subgroups. In conclusion, identifying CHG as a risk factor for DM and elucidating this relationship has significant clinical implications. Incorporating CHG into routine clinical assessment may help clinicians optimize risk stratification and management strategies, enabling timely and personalized interventions. Increasing regular physical activity and improving dietary patterns (such as reducing TC and FPG levels) may help reduce the incidence of DM and ultimately alleviate the public health burden.

Additionally, the aforementioned two cross-sectional studies only assessed the discriminative ability of the CHG index for DM. The study by Li et al. indicated that the AUC value for CHG in diagnosing DM was 0.825 (95% CI: 0.812–0.839)(29). Another study involving U.S. adults found that the AUC value for CHG in DM diagnosis was 0.721 (30). Similarly, a cross-sectional study involving 9,704 participants aged 35 to 65 confirmed that CHG has a high discriminative ability for DM, with an AUC value of 0.864 (95% CI: 0.857–0.871) (26). Furthermore, the AUC of CHG in the present study (0.7377) was lower than those reported in previous studies. This discrepancy is likely attributable to the fundamental difference between cross-sectional designs—which evaluate the diagnostic value of CHG for prevalent DM—and longitudinal designs, which assess its predictive value for incident DM. Our study evaluated the predictive value of CHG for future DM risk over the next five years through ROC curve analysis. In addition, the results indicated that the AUC and Youden index of CHG were both higher than any of its individual components (TC, FPG, and HDL-c). These results suggest that as a novel, clinically accessible, and reproducible composite indicator, CHG has the potential to serve as an early identification marker for high-risk populations of DM and provides important clinical reference value for developing effective intervention measures to reduce DM incidence. However, it should be noted that at the Youden-optimal cutoff (CHG = 5.2366), the sensitivity of CHG was 54.63% with a specificity of 81.61%, indicating that while CHG effectively identifies high-risk individuals with relatively few false positives, approximately 45% of future DM cases may be missed when applied as a single screening threshold. The Youden index optimizes the balance between sensitivity and specificity, but may not be the most appropriate operating point in all clinical contexts. In settings where early case detection is prioritized, a lower CHG threshold favoring higher sensitivity should be considered, accepting a corresponding increase in false positives. Conversely, the relatively high specificity at the current cutoff makes CHG particularly suitable for risk stratification—identifying individuals at elevated risk while minimizing unnecessary follow-up. Therefore, the optimal decision threshold should be selected by weighing clinical objectives, available resources, and the costs of follow-up and intervention. Future studies could explore the integration of CHG with traditional risk factors and emerging biomarkers — such as TG), the atherogenic index of plasma (AIP), the triglyceride-glucose (TyG) index, and the homeostatic model assessment of insulin resistance (HOMA-IR) — within a machine learning framework, with the aim of developing more parsimonious and efficient risk prediction models to further enhance early screening capacity for individuals at high risk of DM.

Excess circulating TC can lead to intracellular cholesterol accumulation in liver and skeletal muscle cells, disrupting insulin receptor signaling pathways and promoting systemic IR (20, 21, 45). Low HDL-c, by reducing ApoA-I availability, impairs ABCA1-dependent reverse cholesterol transport, thereby perpetuating cellular cholesterol overload and its metabolic consequences (22, 46). Within this lipotoxic milieu, small but sustained rises in FPG may indicate early β-cell compensatory exhaustion (47, 48). Thus, CHG integrates these three interrelated components—elevated TC, reduced HDL-c, and elevated FPG—which converge to promote insulin resistance, impaired lipid metabolism, and β-cell dysfunction, collectively driving DM development (25).

This study has several important strengths: (i) Prior cross-sectional studies have demonstrated an association between CHG and prevalent DM in Chinese and U.S. populations. The present retrospective cohort study extends this cross-sectional evidence by longitudinally examining the association between CHG and incident DM in a real-world health-examination cohort, providing stronger evidence for a temporal relationship. CHG was analyzed as both a continuous and categorical variable to minimize information loss, thus providing a more comprehensive and accurate assessment of its association with DM risk. (ii) ROC curve analysis showed that compared to other single indicators (TC, FPG, and HDL-c), CHG demonstrated higher predictive value for DM risk. (iii) Multiple imputation methods were employed to handle missing data, significantly enhancing statistical power and reducing bias due to missing covariate information. (iv) To verify the robustness of our results, multiple sensitivity analyses were conducted: converting CHG into categorical variables; transforming continuous covariates into curves and reincorporating them into the GAM; evaluating potential bias from unobserved and unknown confounding factors through E-value assessment; and reassessing the associations after excluding subjects with obesity (BMI≥28 kg/m²), HTN, or smoking.

However, this study has several limitations that need to be addressed. First, the study population was limited to Chinese subjects, which restricts the external validity of the findings across different ethnic groups or geographic regions, necessitating validation in more heterogeneous populations. Second, only baseline measurements of CHG and other relevant parameters were collected, without evaluating the longitudinal changes of CHG over time. Therefore, future research should collect more comprehensive longitudinal CHG data. Additionally, due to the retrospective cohort design, there were limitations in adjusting for potential confounding factors such as dietary habits and insulin levels. To address this limitation, this study calculated E-values to assess the impact of unknown and unmeasured confounding factors, with results suggesting these factors were unlikely to significantly affect the study conclusions. Subsequent studies should include more relevant parameters to more comprehensively evaluate the relationship between CHG and DM and validate our findings. Additionally, due to individual variability in follow-up visit timing and frequency, not all participants completed the full five-year follow-up period. Although Cox proportional hazards models incorporating each participant’s actual follow-up time were used to minimize the impact of unequal follow-up duration, the possibility of informative censoring—whereby censoring may be related to outcome risk—cannot be entirely excluded, potentially diluting the observed association between CHG and DM. Future prospective cohort studies with more standardized and complete follow-up protocols are warranted to validate these findings. Furthermore, the present study focused on evaluating the independent longitudinal association between CHG as a single composite biomarker and incident DM, rather than developing a high-performance multivariable clinical prediction model. Accordingly, the AUC of CHG (0.7377, 95% CI: 0.7069–0.7685) should be interpreted as a performance benchmark for a single, unadjusted composite index, rather than the ceiling of its predictive potential. Future studies are warranted to integrate CHG with other established risk factors—such as the TyG index, HOMA-IR, HbA1c, age, BMI, and blood pressure—into multivariable prediction models to further enhance discriminative performance and develop a simplified, accessible, and higher-performance clinical prediction tool for DM. Finally, it should be emphasized that this cohort analysis established an independent association between CHG and DM incidence but cannot determine causality.

## Conclusion

This study found an independent positive relationship between CHG and DM risk. Additionally, ROC curve analysis indicated that compared to single indicators (TC, FPG, and HDL-c), CHG demonstrated superior predictive value for DM risk. As a simple and easily obtainable composite indicator, CHG shows promise as an early identification marker for individuals at high risk of DM, providing new insights for optimizing clinical DM prevention and management. Future studies should further explore the incremental predictive value of CHG within multivariable prediction frameworks—by integrating it with established risk factors such as the TyG index, AIP, or HbA1c—to develop a higher-performance and readily applicable clinical prediction model for DM.

## Data Availability

The data analyzed in this study is subject to the following licenses/restrictions: In accordance with the data confidentiality agreement established at the outset of this research, the dataset is not available for public access. Requests to access these datasets should be directed to Researchers who require access to these data for scholarly purposes may contact the corresponding author by email (Hanyong511023@163.com) to submit a formal request.

## Glossary

WC: Waist Circumference
AST: Aspartate Aminotransferase
ALT: Alanine Aminotransferase
CRP: C-Reactive Protein
GGT: Gamma-Glutamyl Transferase
HbA1c: Hemoglobin A1c
Scr: Serum Creatinine
DLP: Dyslipidemia
HTN: Hypertension
HTN-MED: Antihypertensive Medication
DLP-MED: Antihyperlipidemic Medication
TC: Total Cholesterol
LDL-c: Low-Density Lipoprotein Cholesterol
HDL-c: High-Density Lipoprotein Cholesterol
TG: Triglycerides
FPG: Fasting Plasma Glucose
CHG: Total Cholesterol, High-Density Lipoprotein, and Glucose
DM: Diabetes Mellitus
ROC: Receiver Operating Characteristic
IR: Insulin Resistance
AIP: Atherogenic Index of Plasma
TyG: Triglyceride-Glucose Index
HOMA-IR: Homeostatic Model Assessment of Insulin Resistance
