# Supplementary material for: Association of a novel index, the total cholesterol–high-density lipoprotein–glucose index, with diabetes mellitus in Chinese adults: a retrospective cohort study
Source: Front Endocrinol (Lausanne). 2026 Apr 1;17:1774965. doi: 10.3389/fendo.2026.1774965 (PMC13079135; doi:10.3389/fendo.2026.1774965)
Supplement: Supplementary file 1 [file Table1.docx]

**Association of a novel index, the Total Cholesterol–High-Density Lipoprotein–Glucose (CHG) Index, with diabetes mellitus in Chinese adults: A retrospective cohort study**

**Running title:** CHG and DM

**Yajing Gao^1^*, Chuang Gao^2^, Jiaqian Zhu^3,4#^, Yong Han ^5#^**

^1^Department of Anesthesiology, Shenzhen Maternity and Child Healthcare Hospital, Women and Children's Medical Center, Southern Medical University, Shenzhen, Guangdong Province, China.

^2^Department of Emergency, Shenzhen Dapeng New District Kuichong People's Hospital, Shenzhen 518000, Guangdong Province, China.

^3^Department of Neurology, Yiwu Central Hospital, 699 Jiangdong Road, Yiwu, 322000, Zhejiang, China.

^4^School of Medicine, Shenzhen University, The First Affiliated Hospital of Shenzhen University, Shenzhen Second People's Hospital, Shenzhen 518035, Guangdong Province, China.

^5^Department of Emergency, Shenzhen Second People's Hospital, The First Affiliated Hospital of Shenzhen University, Shenzhen 518035, Guangdong Province, China.

**Corresponding author**

Jiaqian Zhu

Department of Neurology, Yiwu Central Hospital

699 Jiangdong Road, Yiwu,

322000, Zhejiang,

Zhejiang Province,

China.

Email: zhujiaqian1122@163.com

**Corresponding author**

Yong Han

Department of Emergency, Shenzhen Second People's Hospital

No.3002 Sungang West Road, Futian District,

Shenzhen 518035,

Guangdong Province,

China.

Email: Hanyong511023@163.com

**Table S1. collinearity screening**

|  | Step 1 | Step 2 | Step 3 | Step 4 | Step 5 | Step 6 |
| --- | --- | --- | --- | --- | --- | --- |
| CHG | 45.5 | 45.5 | 45.5 | 34 | 4.8 | 4.8 |
| sex | 2 | 2 | 2 | 2 | 2 | 1.7 |
| SBP | 3.2 | 3.2 | 3.2 | 3.2 | 3.2 | 3.2 |
| Age | 1.3 | 1.3 | 1.3 | 1.3 | 1.3 | 1.3 |
| DBP | 3.1 | 3.1 | 3.1 | 3.1 | 3.1 | 3.1 |
| BMI | 4.2 | 4.2 | 4.2 | 4.2 | 4.2 | 1.6 |
| WC | 5.2 | 5.2 | 5.2 | 5.2 | 5.2 | NA |
| Smoking | 1 | 1 | 1 | 1 | 1 | 1 |
| LDL-c | 35.6 | 35.6 | 35.6 | 8.6 | 2.3 | 2.3 |
| TG | 6.3 | 6.3 | 6.3 | 2.9 | 1.8 | 1.8 |
| ALT | 1.9 | 1.9 | 1.9 | 1.9 | 1.9 | 1.9 |
| DLP | 1.3 | 1.3 | 1.3 | 1.3 | 1.3 | 1.3 |
| HTN | 4.5 | 4.5 | 4.5 | 4.5 | 4.5 | 4.5 |
| AST | 2.4 | 2.4 | 2.4 | 2.4 | 2.4 | 2.4 |
| HTN_MED | 4.5 | 4.5 | 4.5 | 4.5 | 4.5 | 4.5 |
| DLP_MED | 1.3 | 1.3 | 1.3 | 1.3 | 1.3 | 1.3 |
| GGT | 1.4 | 1.4 | 1.4 | 1.4 | 1.4 | 1.4 |
| Physical Activity | 1.1 | 1.1 | 1.1 | 1.1 | 1.1 | 1.1 |
| HbA1c | Inf | 4.8 | 4.8 | 3.9 | 1.7 | 1.7 |
| CRP | Inf | Inf | 1.1 | 1.1 | 1.1 | 1.1 |

**Variables excluded from collinearity screening: WC**

Continuous variables were presented either as mean ± standard deviation or as median with interquartile range, depending on data distribution. Categorical data were reported as counts and percentages. Abbreviations: Body Mass Index (BMI), Waist Circumference (WC), Total Cholesterol (TC), Low-Density Lipoprotein cholesterol (LDL-c), High-Density Lipoprotein cholesterol (HDL-c), Triglycerides (TG), Fasting Plasma Glucose (FPG), Aspartate Aminotransferase (AST), Alanine Aminotransferase (ALT), C-Reactive Protein (CRP), Gamma-Glutamyl Transferase (GGT), Hemoglobin A1c (HBA1C), Serum creatinine (Scr), Dyslipidemia (DLP), Hypertension (HTN), Hypertension Medication (HTN-MED), Number(N), Dyslipidemia Medication (DLP-MED).

**Table S2. Factors influencing the risk of DM analyzed by univariate Cox proportional hazards regression**

| Variables | Characteristics | HR (95% CI), P |
| --- | --- | --- |
| Sex |  |  |
| Female | 2218 (25.079%) | Ref |
| Male | 6626 (74.921%) | 1.363 (1.035, 1.795) 0.027 |
| Age (years) | 41.819 ± 8.589 | 1.067 (1.055, 1.079) <0.001 |
| SBP (mmHg) | 117.026 ± 12.573 | 1.032 (1.025, 1.039) <0.001 |
| DBP (mmHg) | 76.118 ± 8.119 | 1.051 (1.038, 1.063) <0.001 |
| BMI (kg/m²) | 26.088 ± 3.874 | 1.166 (1.145, 1.188) <0.001 |
| WC (cm) | 91.241 ± 12.173 | 1.061 (1.054, 1.069) <0.001 |
| Smoking |  |  |
| No | 8158 (92.243%) | Ref |
| Yes | 686 (7.757%) | 0.739 (0.470, 1.161) 0.190 |
| TC (mg/dL) | 197.432 ± 36.731 | 1.003 (1.000, 1.006) 0.031 |
| LDL-c (mg/dL) | 123.304 ± 33.670 | 1.003 (1.000, 1.006) 0.084 |
| HDL-c (mg/dL) | 48.959 ± 13.187 | 0.971 (0.962, 0.980) <0.001 |
| TG (mg/dL) | 127.280 ± 80.971 | 1.001 (1.001, 1.002) <0.001 |
| FPG (mg/dL) | 86.884 ± 8.663 | 1.104 (1.093, 1.114) <0.001 |
| AST (U/L) | 29.314 ± 11.618 | 1.010 (1.006, 1.014) <0.001 |
| DLP |  |  |
| No | 6413 (72.512%) | Ref |
| Yes | 2431 (27.488%) | 2.410 (1.933, 3.004) <0.001 |
| HTN |  |  |
| No | 7951 (89.903%) | Ref |
| Yes | 893 (10.097%) | 4.147 (3.253, 5.287) <0.001 |
| ALT (U/L) | 39.894 ± 20.620 | 1.012 (1.009, 1.014) <0.001 |
| HTN_MED |  |  |
| No | 7959 (89.993%) | Ref |
| Yes | 885 (10.007%) | 4.169 (3.264, 5.326) <0.001 |
| DLP-MED |  |  |
| No | 8074 (91.294%) | Ref |
| Yes | 770 (8.706%) | 2.378 (1.766, 3.200) <0.001 |
| CRP (mg/dL) | 2.400 ± 5.052 | 1.011 (0.999, 1.024) 0.080 |
| GGT(U/L) | 27.00 (19.00-39.00) | 1.008 (1.006, 1.010) <0.001 |
| Physical Activity |  |  |
| Sedentary | 1915 (21.653%) | Ref |
| Light activity | 3357 (37.958%) | 0.841 (0.643, 1.098) 0.203 |
| Moderate activity | 2804 (31.705%) | 0.610 (0.451, 0.823) 0.001 |
| Vigorous activity | 768 (8.684%) | 0.664 (0.417, 1.057) 0.084 |
| HbA1c (%) | 5.294 ± 0.267 | 24.539 (17.901, 33.639) <0.001 |
| Scr (μmol/L) | 70.01 ± 15.33 | 1.008 (1.001, 1.029) <0.001 |
| CHG | 5.18 ± 0.35 | 1.196 (1.157, 1.236) <0.001 |

BMI, Body Mass Index; WC, Waist Circumference; TC, Total Cholesterol; LDL-c, Low-Density Lipoprotein Cholesterol; HDL-c, High-Density Lipoprotein Cholesterol; TG, Triglycerides; FPG, Fasting Plasma Glucose; AST, Aspartate Aminotransferase; ALT, Alanine Aminotransferase; CRP, C-Reactive Protein; GGT, Gamma-Glutamyl Transferase; HbA1c, Hemoglobin A1c; Scr, Serum Creatinine; SBP, Systolic Blood Pressure; DBP, Diastolic Blood Pressure; DLP, Dyslipidemia; HTN, Hypertension; HTN-MED, Antihypertensive Medication; DLP-MED, Antihyperlipidemic Medication; CHG, Total Cholesterol, High-Density Lipoprotein, and Glucose Index; DM, Diabetes Mellitus; IR, Insulin Resistance; ROC, Receiver Operating Characteristic; AUC, Area Under the Curve; HR, Hazard Ratio; CI, Confidence Interval; GAM, Generalized Additive Model; VIF, Variance Inflation Factor; IQR, Interquartile Range; SD, Standard Deviation; MAR, Missing at Random; N, Number; Ref, Reference.

**Table S3. Stratified associations between CHG(per 0.1-unit) and DM by age, sex, SBP, DBP, physical activity, DLP, and drinking status.**

| Characteristic | No of participants | DM events(N) | HR (95%CI) P value P for interaction |
| --- | --- | --- | --- |
| Age(years) |  |  | 0.7498 |
| <40 | 3296 | 63 | 1.148 (1.063, 1.240) <0.001 |
| 40-50 | 3365 | 137 | 1.141 (1.074, 1.212) <0.001 |
| ≥50 | 1728 | 124 | 1.169 (1.094, 1.248) <0.001 |
| Sex |  |  | 0.2942 |
| Male | 6626 | 261 | 1.174 (1.115, 1.236) <0.001 |
| Female | 2218 | 63 | 1.121 (1.035, 1.213) 0.005 |
| SBP (mmHg) |  |  | 0.8887 |
| <140 | 8207 | 270 | 1.160 (1.106, 1.217) <0.001 |
| ≥140 | 637 | 54 | 1.151 (1.042, 1.272) 0.005 |
| DBP (mmHg) |  |  | 0.3120 |
| <90 | 8096 | 266 | 1.150 (1.097, 1.206) <0.001 |
| ≥90 | 748 | 58 | 1.213 (1.095, 1.344) <0.001 |
| Physical Activity |  |  | 0.5323 |
| **Sedentary** | 1915 | 91 | 1.163 (1.082, 1.251) <0.001 |
| **Light** | 3357 | 131 | 1.190 (1.118, 1.266) <0.001 |
| Moderate | 2804 | 80 | 1.116 (1.036, 1.201) 0.005 |
| Vigorous | 768 | 22 | 1.137 (0.995, 1.299) 0.058 |
| DLP |  |  | 0.3962 |
| No | 6413 | 183 | 1.161 (1.101, 1.225) <0.001 |
| Yes | 2431 | 141 | 1.125 (1.054, 1.201) <0.001 |
| Drinking |  |  | 0.7430 |
| Never | 398 | 27 | 1.155 (0.951, 1.403) 0.147 |
| Current | 1259 | 49 | 1.124 (1.026, 1.232) 0.012 |
| Ever | 7387 | 248 | 1.168 (1.111, 1.227) <0.001 |

Note 1: Above model adjusted for age, BMI, drinking status, ALT, TG, HbA1c, physical activity, DBP, Scr, smoking status, AST, HTN, DLP-MED, and SBP were adjusted.

Note 2: In each case, the model is not adjusted for the stratification variable.

HR, Hazard ratios; CI: confidence, Ref: reference.
